# Caspase inhibitor attenuates the shape changes in the alveolar ridge following tooth extraction: A pilot study in rats

**DOI:** 10.1111/jre.12798

**Published:** 2020-09-16

**Authors:** Uwe Yacine Schwarze, Franz‐Josef Strauss, Reinhard Gruber

**Affiliations:** ^1^ Department of Oral Biology Medical University of Vienna Vienna Austria; ^2^ Division of Oral Surgery and Orthodontics Department of Dental Medicine and Oral Health Medical University of Graz Graz Austria; ^3^ Austrian Cluster for Tissue Regeneration Vienna Austria; ^4^ Clinic of Reconstructive Dentistry Center of Dental Medicine University of Zurich Zurich Switzerland; ^5^ Department of Conservative Dentistry Faculty of Dentistry University of Chile Santiago Chile; ^6^ Department of Periodontology School of Dental Medicine University of Bern Bern Switzerland

**Keywords:** atrophy, buccal bone, caspase inhibitor, osteocytes, resorption, ridge preservation, socket preservation, tooth extraction

## Abstract

**Objective:**

The aim of the study was to determine whether the inhibition of apoptosis via pan‐caspase inhibitors can attenuate the changes in the alveolar ridge upon tooth extraction.

**Background:**

Cells undergoing apoptosis might play a central role in the onset of alveolar bone resorption and the ensuing bone atrophy following tooth extraction. Caspases are proteases that regulate apoptotic cell death. It is, therefore, reasonable to hypothesize that blocking apoptosis with pan‐caspase inhibitors attenuates the changes in the alveolar ridge following tooth extraction.

**Methods:**

In 16 inbred rats, the mandibular first (M1) and second (M2) molars of one side were extracted. Following random allocation, the rats received either a cell‐permeable pan‐caspase inhibitor or diluent. After a healing period of 10 days, changes in shape and height of the alveolar ridge were examined using geometric morphometrics and linear measurements based on micro‐computed tomography.

**Results:**

Geometric morphometric analysis revealed that the pan‐caspase inhibitor prevented major shape changes of the alveolar ridge following M1 tooth extraction (*P* < .05). Furthermore, linear measurements confirmed that the pan‐caspase inhibitor significantly prevented the atrophy of the alveolar ridge height following M1 tooth extraction compared to the diluent controls (−0.53 mm vs −0.24 mm; *P* = .012). M2 tooth extraction caused no shape changes of the alveolar ridge, and thus, the pan‐caspase inhibitor group did not differ from the control group (−0.14 mm vs −0.05 mm; P = .931).

**Conclusions:**

These findings suggest that the inhibition of apoptosis may attenuate shape changes of the alveolar ridge following M1 tooth extraction in rodents.

## 1 INTRODUCTION

Following tooth extraction, the alveolar bone undergoes evident resorption.^1^ Due to this catabolic event, bone augmentation often becomes mandatory to allow implants to be placed in a prosthetically driven position. Consequently, there is intense research focused on maintaining the dimensions of alveolar bone after tooth extraction. This can be attained to some extent by performing a procedure termed “alveolar ridge preservation.”^2^ The most common technique is to fill up the extraction socket with a bone substitute material and cover it with a resorbable membrane.^2^ This procedure, however, does not prevent dimensional alterations but does limit the extent to which resorption occurs.^2^ As a result, there is a clear demand to identify new strategies to prevent the resorption of the alveolar ridge. In this context, a better understanding of the underlying molecular and cellular mechanisms that cause alveolar bone resorption may provide a scientific basis to develop such strategies.

Tooth extraction, similar to other injuries, may induce apoptosis, a sequence of molecular events controlling cell death.^3^ Apoptotic cells are present in the periodontal ligament following tooth extraction in rats.^4^, ^5^, ^6^ Although apoptosis is a rare event in osteocytes after atraumatic tooth extraction as compared to osteotomies^4^ or following orthodontic tooth movement,^7^ these apoptotic cells may release signals that ultimately trigger and coordinate tissue repair. This is of particular importance since dying osteocytes elicit the formation of osteoclasts and consequently bone resorption.^6^, ^7^, ^8^ Furthermore, it appears that the invasiveness of the surgical procedure determines the degree of osteocytes’ apoptosis and consequently alveolar bone resorption. This suggests that apoptosis may play a major role in the subsequent catabolic changes of the alveolar ridge following tooth extraction.

Such a hypothesis prompted us to investigate whether a pharmacologic therapy based on a cell‐permeable inhibitor of caspases can prevent catabolic changes after tooth extraction. Caspases belong to a family of proteases that regulate apoptotic cell death.^8^, ^9^ Inhibition of apoptosis using a pan‐caspase inhibitor reduces the particle‐induced osteolysis in mice^8^ and prevents the trabecular bone loss caused by unloading.^9^ Moreover, the increase in bone resorption does not occur in ovariectomized mice treated with a pan‐caspase inhibitor.^10^ There is thus a potential of pan‐caspase inhibitors to attenuate catabolic changes of alveolar bone following tooth extraction. The aim of the present study was, therefore, to determine whether the use of a pan‐caspase inhibitor can attenuate structural catabolic changes in the alveolar ridge after tooth extraction in rats.

## 2 MATERIAL AND METHODS

### 2.1 Animals and surgery

Ethical approval for this study was granted by the Federal Ministry for Science, Research and Economy (GZ BMWFW‐66.009/0020‐WF/V/3b/2017), and research was conducted according to the ARRIVE guidelines. Protocols, handling, and care of the mice conformed to the Austrian federal law for animal protection. Sample size calculation was based on a previous study.^11^ In brief, we expected a 15% effect of the difference between the groups. Considering a power 80%, a type I error rate of 5%, and six degrees of freedom, seven rats per group were necessary and one extra rat per group was added in the event of an unexpected loss. Consequently, a total of 16 inbred male rats, 4 weeks of age, underwent tooth extraction of the right mandibular first molar (M1) and the left mandibular second molar (M2). Anesthesia and analgesia were given i.p. injecting ketamine (100 mg/kg), xylazine (5 mg/kg), and piritramide (3 mg/kg). Enrofloxacin (Baytril 2.5% 15 mg/kg/d) was provided in 5% dextrose water against infections. Following tooth extraction, the animals were randomly allocated to receive for 10 consecutive days either a subcutaneous cell‐permeable pan‐caspase inhibitor (Z‐Val‐Ala‐DL‐Asp‐fluoromethylketone (Bachem Distribution, Services GmbH, Hegenheimer Strasse 5, D‐79576 Weil am Rhein); z‐VAD‐fmk; 10 mg/kg/d; CASPASE group) or a subcutaneous diluent (CONTROL group) consisting of equivolumetric dimethyl sulfoxide and 0.9% saline solution. Rats were randomly assigned with GraphPad QuickCalcs for treatment groups (www.graphpad.com). In addition, in the CASPASE group, a sponge (3 × 3 × 2 mm; MS002 Spongostan Standard by Ferrosan Soeborg, Denmark) soaked in 1 µg/mL z‐VAD‐fmk was adapted and placed in the extraction sockets for 2 minutes. This local one‐time procedure was supposed to prevent cell death until the absorption of the systemic pan‐caspase inhibitor. Considering previous studies^12^ indicating bone gain after tooth extraction at day 14 in rats, the euthanasia was performed on day 11 and with an overdose of pentobarbital (300 mg/kg i.c.).

### 2.2 Micro‐CT analysis

Mandibles samples extracted and fixed in phosphate‐buffered formaldehyde (Roti‐Histofix 4%, Carl Roth). µCT images taken at 90 kV/200 µA, isotropic resolution of 17.2 µm, and an integration time of 500 ms (µCT 50, Scanco Medical AG) were reconstructed using amira 6.2 (Thermo Fisher Scientific).

### 2.3 Generalized Procrustes analysis and principal component analysis

**Figure 1:**
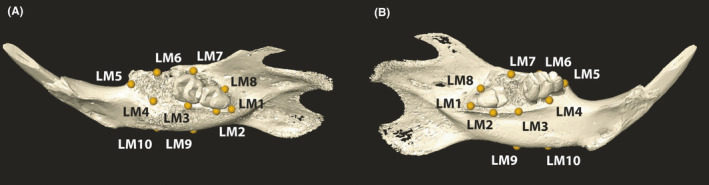
Landmarks on right and left mandible. A, Right mandible with extracted M1. B, Left mandible with extracted M2. LM1 and LM5 are the most distal and mesial points of the alveolar ridge, respectively. LM2‐LM4 and LM6‐LM8 represent the lingual and buccal mid points of the alveolar bone ridge. LM9 and LM10 are the lowest points of the mandible at the position of M2 and M1, respectively. The linear distance LM4‐LM10 and LM3‐LM9 represent M1 and M2 alveolar ridge heights and are depicted in Figure 3A,B and Table 1

Bony surfaces were generated with Amira using the half‐maximum height value as described by Weber and Bookstein 2011. Ten landmarks on characteristic anatomical sites were set (Figure 1). A generalized Procrustes analysis (GPA) was performed to superimpose and unify the landmarks in all samples with respect to location, size, and rotation (EVAN Toolbox 1.72 EVAN‐Society e.V., Vienna, Austria, 2014) being visualized (Public beta © 2005 Dennis E. Slice). For comparing the shapes of the alveolar bone, a principal component analysis (PCA) based on eight landmarks was performed. The differences in shapes are represented by data plotted on a principal component axis.^13^ Statistical significance is given when data points of groups (≥3 Individuals/group) do not overlap. The shape difference distinguishing the groups were visualized by relative warps using extreme scores (−0.25 and 0.25) on the principal component two. This analysis identified regions with the strongest shape difference which were further used for linear measurements.

### 2.4 Linear measurements of alveolar bone high

We calculated the distances between the lingual^12^ alveolar ridge and the lowest part of the mandible at the respective tooth position (LM4‐LM10, LM3‐LM9). The mandibular first (M1) and second (M2) molars of one side were extracted, and the contralateral pristine bone sites were used as reference. When µCT images revealed remnants of teeth in the extraction alveolus, we excluded the values; according to that method, four extraction alveoli were excluded. Lingual alveolar crest distance of M1 was size‐corrected by dividing the distance by the centroid size. To further address the ridge resorption difference, we subtracted the ridge height of the pristine and extraction side to obtain a delta.

### 2.5 Statistical analysis

We performed the nonparametric Mann‐Whitney test for all variables due to the small sample size even though some variables passed the Shapiro‐Wilk test for normality. Tests and graphics were performed using graphpad prism version 8.3.15. Owing to the pilot nature of the study, the sample size was chosen based on experience from previous studies^11^ to balance the ability to measure significant differences while reducing the number of animals used. Significance was set at *P* < .05.

## 3 RESULTS

### 3.1 Caspase inhibitor attenuates shape changes of the mandible after M1 tooth extraction

**Figure 2:**
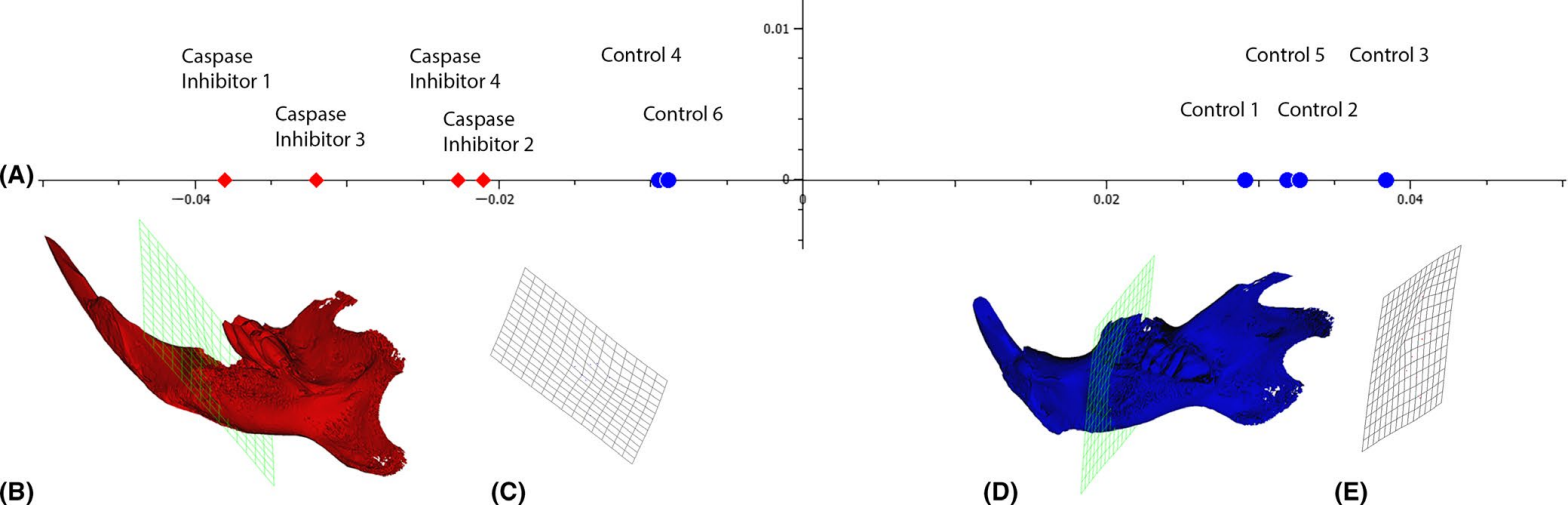
Principal component analysis (PCA) in shape space of PC2. A) Red diamonds and blue dots represent the individuals in shape space of PC2 receiving the caspase inhibitor and diluent, respectively (*P* = .01). B) and C) Relative warps and deformed grids at molar 1 (M1) representing pan‐caspase inhibitor at −0.25. D) and E) controls at 0.25. The relative warps (B, D) and grids (C,E) reveal a difference in the ridge height at position M1 with a longer ridge height and a rotation upwards in the caspase inhibitor group

To identify possible shape changes caused by the pan‐caspase inhibitor, a PCA on the extraction sockets was performed. The PCA revealed that the caspase inhibitor significantly affected shape changes at M1, indicated by the separation of the groups along the projected axis (Figure 2A, *P* = .005). These differences were not observed at M2 (data not shown). The relative warps (Figure 2B,D) and deformation grids (Figure 2C,E) revealed the direction of how the pan‐caspase inhibitor altered the shape at M1 extraction sites. The caspase inhibitor reduced the lingual bone atrophy and the subsequent lingual rotation of the alveolar ridge. Overall, these findings suggest that caspase inhibitor attenuates the catabolic shape changes of the alveolar ridge after M1 tooth extraction.

### 3.2 Caspase inhibitor reduced alveolar ridge atrophy upon tooth extraction

**Figure 3:**
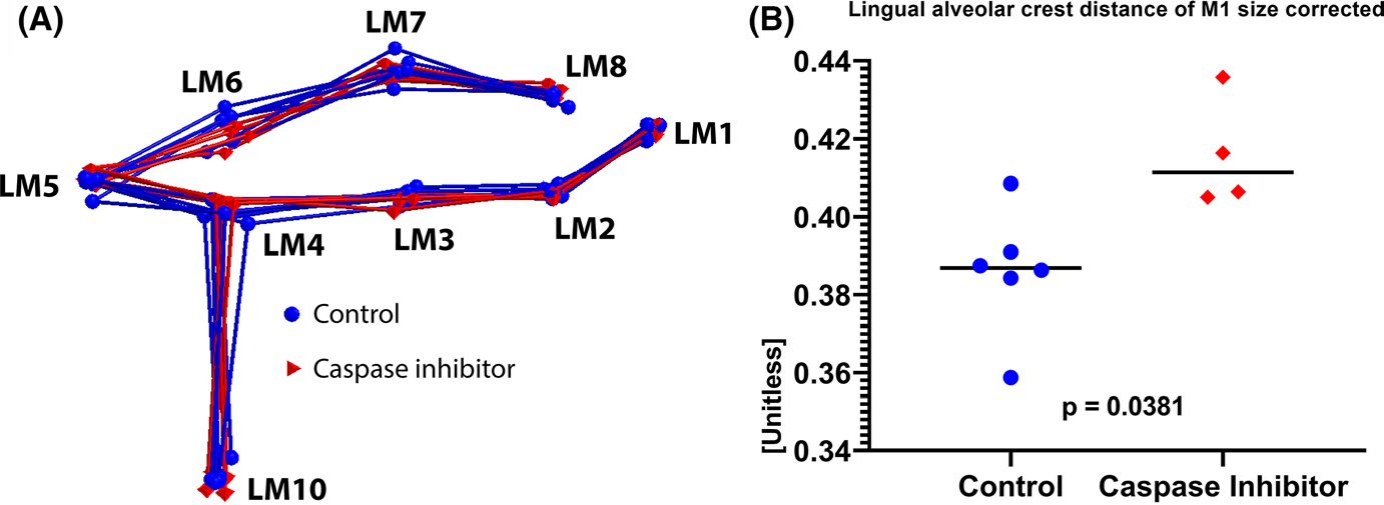
Generalized Procrustes analysis (GPA) and standardized alveolar ridge height. A) The GPA is a superimposition of all the landmarks in which differences such as translation, size, and rotation are standardized. B) The alveolar ridge height distances (LM4‐L10) of the caspase inhibitor group (red diamonds) are relatively larger than the distances of the control group (blue dots); *P* = .0381

**Figure 4:**
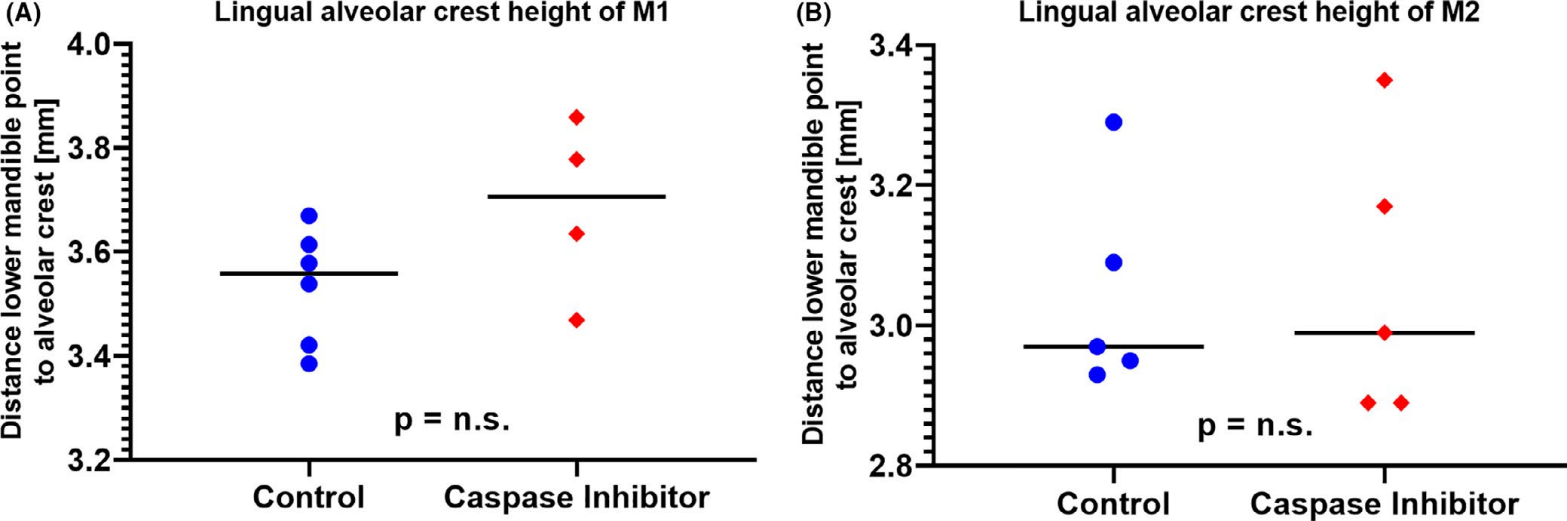
Alveolar ridge height measurements of position M1(LM4‐LM10) (A) and M2 (LM3‐LM9) (B). Absolute and therefore not corrected alveolar ridge height shows no statistical differences either in M1 or M2.

To further examine the effect of caspase inhibitor on bone shape, a GPA was performed. The GPA uses landmarks to superimpose the anatomical sites (Figure 3). Superimposition of nine size‐, rotation‐, and translation‐corrected landmarks exhibited a difference between the caspase inhibitor group and the control group (Figure 3A). The M1‐corrected landmarks (LM4‐LM10) revealed a higher lingual ridge in the caspase inhibitor group compared to the control group (Figure 3B; *P* = .038). Also, uncorrected M1 landmarks (LM4‐L10) exhibited a higher lingual ridge in the caspase inhibitor group, but without reaching significance (*P* = .171; Figure 4A). M2 landmarks (LM3‐LM9) did not differ between the groups, irrespective of the correction (Table 1; Figure 4B).

**Table 1:** Summary of measurements and statistic

	Size‐corrected alveolar ridge distance of M1R (unitless)^a^	PC2 values (unitless)^b^	Alveolar ridge height (mm)^c^				Alveolar atrophy (mm)^d^	
			At M1R extracted (LM4‐LM10)^e^	At M1L pristine (LM4‐LM10)^e^	At M2L extracted (LM3‐LM9)^e^	At M2R pristine (LM3‐LM9)^e^	Delta of M1 (M1R‐M1L)	Delta of M2 (M2L‐M2R)
Effect size	0.0002	0.0003	0.0196	0.2571	0.0277	0.6623	0.011	0.035
*P*‐value	.038	.010	.171	No effect expected	.937	No effect expected	0.012	0.931
CO mean	0.386	0.019	3.510	4.040	3.045	3.183	−0.531	−0.138
Std. error of CO mean	0.007	0.009	0.045	0.048	0.067	0.068	0.039	0.096
PCI mean	0.416	−0.028	3.686	3.924	3.060	3.107	−0.239	−0.047
Std. error of PCI mean	0.007	0.004	0.086	0.055	0.089	0.049	0.051	0.509
Control	0.359	0.029	3.421	4.025	3.288	3.263	−0.604	0.025
	0.384	0.033	3.386	3.888	2.953	3.049	−0.503	−0.096
	0.391	0.038	3.579	4.056	2.926	3.285	−0.477	−0.359
	0.409	−0.009	3.670	4.023	x	x	−0.353	x
	0.387	0.032	3.614	4.255	x	x	−0.640	x
	0.386	−0.009	3.539	4.037	2.966	3.327	−0.498	−0.361
Pan‐caspase inhibitor	0.436	−0.038	3.859	4.079	3.350	3.108	−0.220	0.242
	0.406	−0.021	3.469	3.852	2.887	2.912	−0.383	−0.026
	0.405	−0.032	3.635	3.844	2.995	3.119	−0.209	−0.124
	x	x	x	x	2.894	3.106	x	−0.212
	0.416	−0.023	3.778	3.920	3.175	3.292	−0.142	−0.117

Note

M1R = molar 1 of right mandible; M1L = molar 1 of left mandible. M2L = molar 2 of left mandible; M2R = molar 2 of right mandible; CO = control; PCI = pan‐caspase inhibitor; X = excluded for remnants of tooth in the socket; LM = landmark.

^a^ Size‐corrected alveolar ridge distance of M1R (unitless) is the distance between landmark 4 and landmark 10 divided by the centroid size measured by the generalized Procrustes analysis.

^b^ PC2 values are the principal component 2 shape values as computed in the principal component analysis.

^c^ The alveolar ridge heights are the values between the distances of landmarks 4 and 10 for the molar one and landmarks 3 and 9 for molar to on the right and left side of the mandible.

^d^ Alveolar atrophy is calculated by subtracting the ridge height of the extracted side minus the pristine side for the respective molars.

^e^ Molar one is extracted on the right mandible but pristine on the left and molar 2 vice versa.

**Figure 5:**
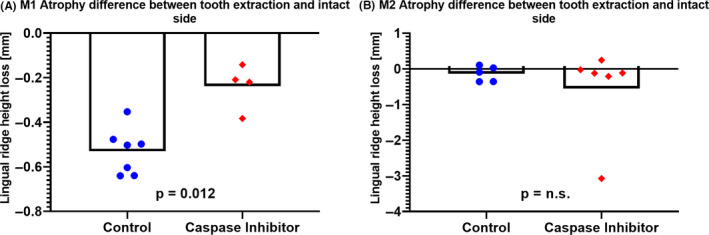
Atrophy differences in molar one (M1) and molar two (M2). The bone atrophy was determined by calculating the difference between the extraction sockets and the pristine corresponding sites. Significant less atrophy was observed for the caspase inhibitor group compared to the control group in M1 (A) but not in M2 (B).

Owing to the length differences of the lingual ridge at M1 extraction sites, we compared the alveolar ridge height between the extraction and the contralateral pristine sites. The pan‐caspase inhibitor significantly attenuated the alveolar ridge atrophy when compared to the control group (−0.24 mm vs −0.53 mm; *P* = .012; Figure 5A). Expectedly, at M2 sites, the lingual ridge height barely changed upon tooth extraction (−0.14 mm vs −0.05 mm; *P* = .931; Figure 5B). These observations indicate that caspase inhibitor is capable of preserving the lingual alveolar ridge upon M1 tooth extraction.

## 4 DISCUSSION

The present study demonstrated that pan‐caspase inhibitors attenuate catabolic changes of the alveolar ridge after M1 tooth extraction. These findings can be explained by the inhibition of apoptosis that might otherwise have caused bone resorption. These biological principles are supported by a previous study showing that the pan‐caspase inhibitor reduces the inflammation‐induced bone resorption in a murine calvaria model.^8^ Similarly, results from another study revealed that pan‐caspase inhibitors were able to attenuate the hindlimb‐induced bone loss in mice.^9^ Thus, the present data support the hypothesis that M1 tooth extraction triggers a local cell apoptosis and the dying cells in turn generate a microenvironment that support osteoclastogenesis leading to bone loss. Conversely, in M2 sites the alveolar ridge barely changed upon tooth extraction. This might be attributed to the M2 anatomical situation where tooth extraction may causeless mechanical stress and apoptosis, resulting in limited bone resorption.

A recent study revealed that bisphosphonates also prevent bone atrophy after the extraction of mandibular first molars in rats.^14^ In that study, buccal and lingual alveolar bone atrophy was significantly reduced after 1 month of healing. Conversely, in our study we observed catabolic changes in alveolar bone at 10 days. However, it should be noted that the anatomical changes in rodent models are subtle and can hardly be localized by linear measurements. Our analyses were therefore based on geometrics morphometrics being widely recognized as robust and sophisticated diagnostic tools for determining shape changes, particularly in anthropology.^15^, ^16^, ^17^ This type of analysis has also been used in dentistry, including orthodontics^18^, ^19^, ^20^ and craniofacial surgery.^21^ In addition, we have recently proposed the use of geometrics morphometrics to characterize skull deformation in sclerostin knockout mice.^11^ Here, geometric morphometrics revealed that caspase inhibitors limited the deformation of the lingual alveolar ridge following M1 tooth extraction. In addition, it showed that M2 tooth extraction causes almost no catabolic changes.

We recognize that this study has a number of limitations. First, this study focused on the structural changes after tooth extraction. In this sense, to which extent apoptotic osteocytes^4^ and periodontal cells^4^, ^5^, ^6^ drive bone resorption upon tooth extraction remains unclear. Considering that both aforementioned cell types can contribute to inflammatory osteolysis of the alveolar bone,^22^, ^23^ future research should provide further insights on the mechanisms and the cell types involved in alveolar bone resorption. Second, caspase‐3 knockout mice exhibit significant bone defects during early development^24^ suggesting that these models are not ideal to study the impact of apoptosis on alveolar ridge atrophy. Third, the small sample size and the exclusion of sockets with teeth remnants limited the power of this pilot study. However, due to ethical reasons we decided to include as few animals as possible. Geometric morphometrics is robust enough to detect small effects even with low sample size thereby reducing the numbers of animals needed. Fourth, whether or not a more traumatic M2 tooth extraction increases the rate of osteocyte's apoptosis and bone atrophy remains to be determined. Fifth, no histology was performed since we could not predict the region of interest before geometric morphometrics. Furthermore, the catabolic changes might have been too small to be identified by histomorphometry. Finally, questions related to the route of administration and the dosing of pan‐caspase inhibitors, as well as the translation into a higher phylogenetic organism, are open and should inspire future research.

## 5 CONCLUSION

The present study suggests that the inhibition of apoptosis via caspase inhibitors may attenuate shape changes of the alveolar ridge following M1 tooth extraction in rats. Our findings support the hypothesis that resorption of the alveolar ridge and the subsequent bone atrophy following tooth extraction is partially controlled by apoptotic mechanisms. Targeting apoptosis might become an interesting therapeutic approach to modulate the dimensional changes of the alveolar bone following tooth extraction.

## CONFLICT OF INTEREST

The project was supported by a grant (15‐244) from the Osteology Foundation, Switzerland. This project was further supported by a grant from Austrian Science Fund (FWF) (4072‐B28). The authors declare no conflict of interest.

## AUTHOR CONTRIBUTIONS

UYS, FJS, and RG conceived and designed the study. UYS and FJS involved in acquisition. UYS performed formal analysis. UYS and RG interpreted the data. UYS and RG wrote the original draft. UYS, FJS, and RG substantively revised. UYS involved in visualization. RG supervised the study. UYS involved in project administration. UYS and RG acquired the funding. All authors have read and approved the submitted version of the manuscript (and any substantially modified version that involves the author's contribution to the study).
